# Overview on the Biochemical Potential of Filamentous Fungi to Degrade Pharmaceutical Compounds

**DOI:** 10.3389/fmicb.2017.01792

**Published:** 2017-09-20

**Authors:** Darío R. Olicón-Hernández, Jesús González-López, Elisabet Aranda

**Affiliations:** ^1^Environmental Microbiology Group, Department of Microbiology, Institute for Water Research, University of Granada Granada, Spain; ^2^Department of Microbiology, Faculty of Pharmacy, University of Granada Granada, Spain

**Keywords:** *Ascomycota*, *Mucoromycotina*, *Basidiomycota*, emerging contaminants, cytochrome P450

## Abstract

Pharmaceuticals represent an immense business with increased demand due to intensive livestock raising and an aging human population, which guarantee the quality of human life and well-being. However, the development of removal technologies for these compounds is not keeping pace with the swift increase in their use. Pharmaceuticals constitute a potential risk group of multiclass chemicals of increasing concern since they are extremely frequent in all environments and have started to exhibit negative effects on micro- and macro-fauna as well as on human health. In this context, fungi are known to be extremely diverse and poorly studied microorganisms despite being well suited for bioremediation processes, taking into account their metabolic and physiological characteristics for the transformation of even highly toxic xenobiotic compounds. Increasing studies indicate that fungi can transform many structures of pharmaceutical compounds, including anti-inflammatories, β-blockers, and antibiotics. This is possible due to different mechanisms in combination with the extracellular and intracellular enzymes, which have broad of biotechnological applications. Thus, fungi and their enzymes could represent a promising tool to deal with this environmental problem. Here, we review the studies performed on pharmaceutical compounds biodegradation by the great diversity of these eukaryotes. We examine the state of the art of the current application of the *Basidiomycota* division, best known in this field, as well as the assembly of novel biodegradation pathways within the *Ascomycota* division and the *Mucoromycotina* subdivision from the standpoint of shared enzymatic systems, particularly for the cytochrome P450 superfamily of enzymes, which appear to be the key enzymes in these catabolic processes. Finally, we discuss the latest advances in the field of genetic engineering for their further application.

## Pharmaceutical compounds in the environment

Pharmaceutical compounds, also referred to as pharmaceutically active compounds (PhACs) regarding specifically the active constituent, are biologically active substances widely used for therapeutic purposes in livestock (Table 1). Thousands of drugs are used globally as a regulated therapeutic or adjuvant element, hundreds more can be purchased without medical supervision and the potential for adverse risk in the environment has not been established (Strauch, 2011). Even more alarming, the uncontrolled use of antibiotics in livestock breeding and aquaculture, which use is, in many parts of the world, much greater than for humans, and which has contributed to their presence in surface ground- and waste-water (Van Boeckel et al., 2015). Only a few effects have been described for PhACs, including bioaccumulation, endocrine disruption, different kinds of diseases, acquisition of antibiotic-resistance gene in bacteria and changes in microbial populations or biomagnifications (Kümmerer, 2009; Czekalski et al., 2014; Barra Caracciolo et al., 2015; Du et al., 2015; Giulivo et al., 2016).

**Table 1:** Pharmaceutical compounds considered emerging contaminants.

**Emerging contaminant**	**Chemical structure**	**M.W. (g/mol)**	**CAS number**	**Solubility in water^a^**	**Origin**
**ILLICIT DRUGS**					
α-methylphenethylamine (Amphetamine) (C_9_H_13_N)	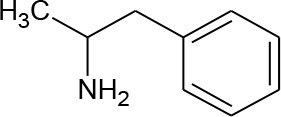	135.21	30-62-9	Slightly	Urban and hospital wastewater
N-methylamphetamine (Methamphetamine) (C_10_H_15_N)	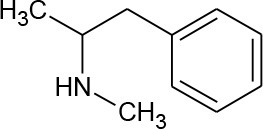	149.23	537-46-2	5 × 10^5^ mg/L at 25°C	
**PHARMACEUTICALS**					
Acetaminophen (Paracetamol) (C_8_H_9_NO_2_)	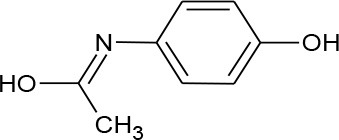	151.165	103-90-2	1.4 × 10^4^ mg/L at 25°C	Human intake and excretion in municipal wastewater, hospitals and pharmaceutical waste, and landfills
Carbamazepine (C_15_H_12_N_2_O)	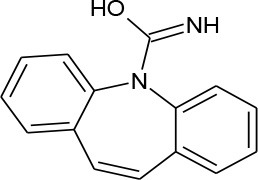	236.274	298-46-4	18 mg/L at 25°C	
Ciprofloxacin (C_17_H_18_FN_3_O_3_)	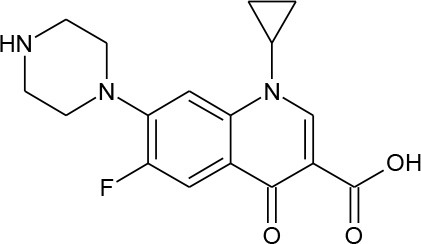	331.347	85,721-33-1	3 × 10^4^ mg/L at 20°C	
Clofibric acid (C_10_H_11_ClO_3_)	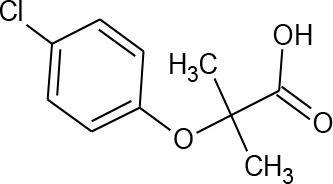	214.645	882-09-7	583 mg/L at 20°C	
Diazepam (C_16_H_13_ClN_2_O)	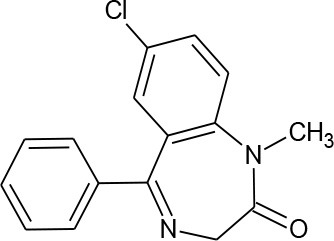	284.743	439-14-5	66 mg/L at 25°C	
Diclofenac (C_14_H_11_Cl_2_NO_2_)	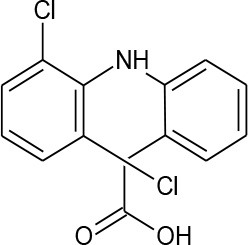	296.147	15,307-86-5	2.37 mg/L at 25°C	
Flumequine (C_14_H_12_FNO_3_)	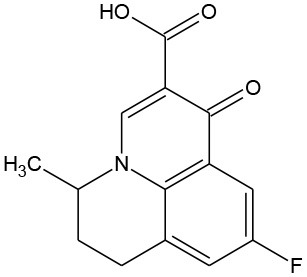	261.252	42,835-25-6	2.19 mg/mL at 25°C	
Furosemide (C_12_H_11_ClN_2_O_5_S)	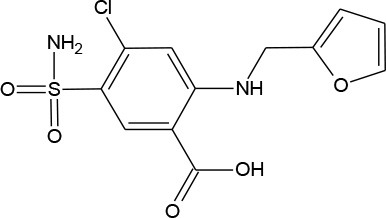	330.739	54-31-9	>1 mg/mL at 25°C	
Gemfibrozil (C_15_H_22_O_3_)	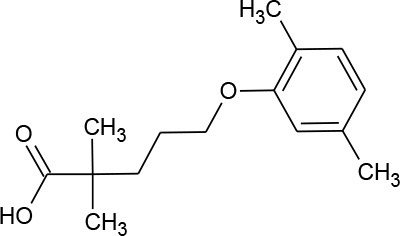	250.338	25,812-30-0	11 mg/L at 25°C	
Ibuprofen (C_13_H_18_O_2_)	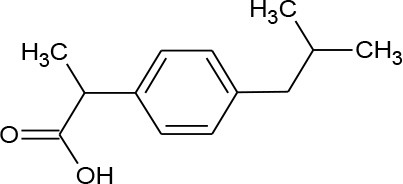	206.285	15,687-27-1	21 mg/L at 25°C	
Metoprolol (C_15_H_25_NO_3_)	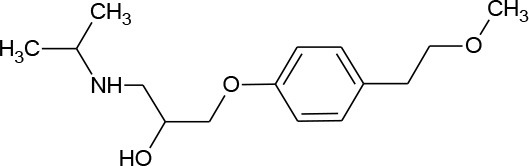	267.369	51384-51-1	1.6 × 10^4^ mg/L at 25°C	
Naproxen (C_14_H_14_O_3_)	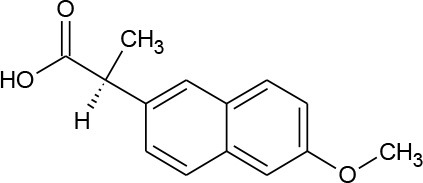	230.263	22,204-53-1	15.9 mg/L at 25°C	
Omeprazole (C_17_H_19_N_3_O_3_S)	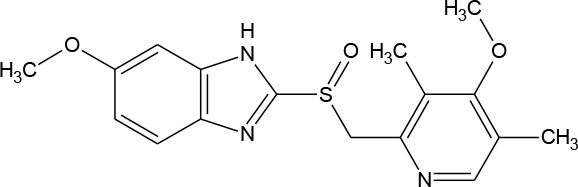	345.417	73,590-58-6	3.54 × 10^−2^ mg/mL at 25°C	
Sulfamethoxazole (C_10_H_11_N_3_O_3_S)	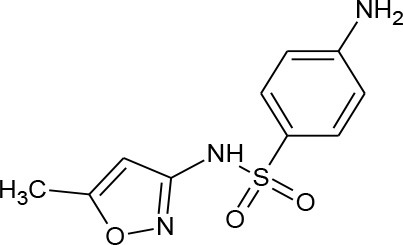	253.276	723-46-6	610 mg/L at 37°C	
Triclosan (C_12_H_7_Cl_3_O_2_)	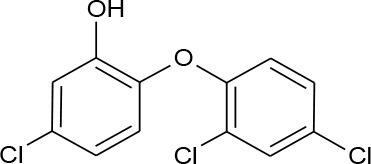	289.536	3,380-34-5	10 mg/L at 20°C	
Trimethoprim (C_14_H_18_N_4_O_3_)	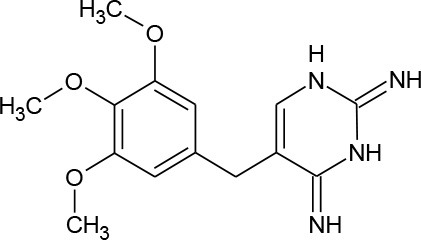	290.323	738-70-5	400 mg/L at 25°C	
Valproic acid (C_8_H_16_O_2_)	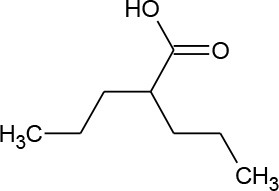	144.214	99-66-1	2.0 × 10^3^ mg/L at 20°C	
Warfarin (C_19_H_16_O_4_)	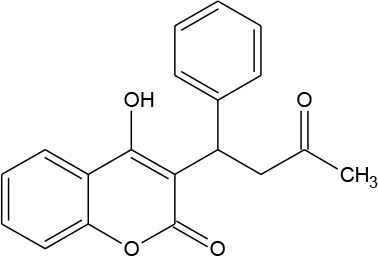	308.333	81-81-2	1.7 × 10^−2^ mg/mL at 20°C	
**PERSONAL-CARE PRODUCTS**					
Benzophenone (C_13_H_10_O)	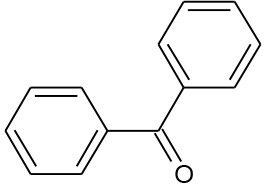	182.222	119-61-9	1.37 × 10^2^ mg/L at 25°C	Domestic and industry effluents.
Ethylhexyl methoxycinnamate (C_18_H_26_O_3_)	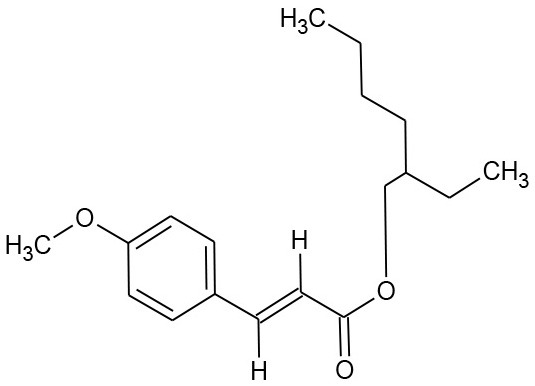	290.403	5466-77-3	0.2 mg/L at 20°C	
Galaxolide (C_18_H_26_O)	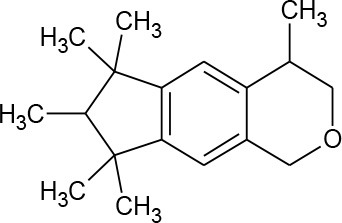	258.405	1,222-05-5	1.75 mg/L at 25°C	
**HORMONES**					
17-beta-estradiol (C_18_H_24_O_2_)	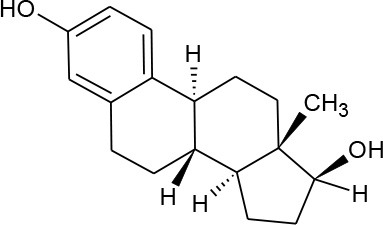	272.388	50-28-2	3.90 mg/L at 27°C	Human and veterinary treatment. Hospital and domestic effluent
Estriol (C_18_H_24_O_3_)	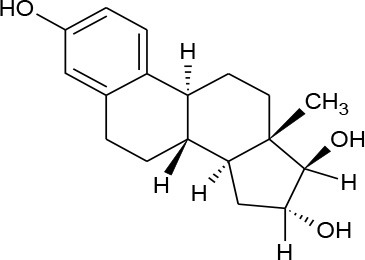	288.387	50-27-1	27.34 mg/L at 25°C	
Estrona(C_18_H_22_O_2_)	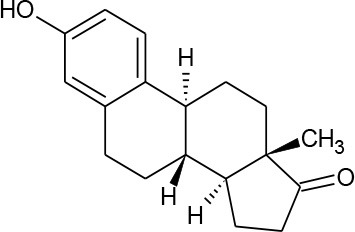	270.372	53-16-7	12.42 mg/L at 25°C	
Mestranol (C_21_H_26_O_2_)	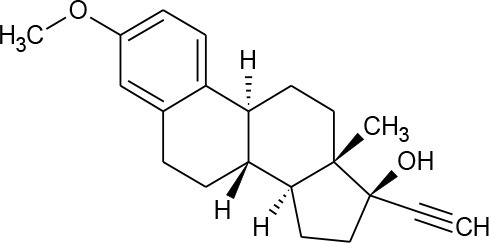	310.437	72-33-3	1.132 mg/L at 25°C	
**OTHERS**					
Bisphenol A(C_15_H_16_O_2_)	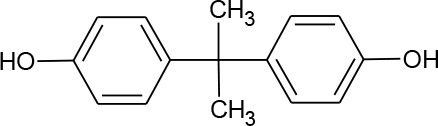	228.291	80-05-7	300 mg/L at 25°C	Various
Nonylphenol(C_15_H_24_O)	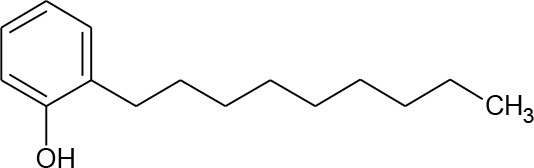	220.356	25,154-52-3	600 mg/L at 25°C	
Titanium dioxide (TiO_2_)	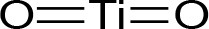	79.865	13,463-67-7	less than 1 mg/mL at 25°C	

a *The data were gathered from the (NCBI) in the PubChem database https://pubchem.ncbi.nlm (Kim et al., 2016)*.

The legislation of the use of PhACs is different in each country and also depends on the range or employment, distribution, and resulting prevalence in the environment. However, the lack of information on long-term risks limits and delays updates in the field of the regulation of PhACs, and therefore are considered emerging contaminants (ECs). There are international control organizations that regulate the use of PhACs among others ECs around the world; for example the joint FAE/WHO Expert Committee on Food Additives (JECFA) constantly evaluates the risk for the use of different PhCs such as derquantel, dexamethasone, testosterone, progesterone and avilamycin, among others, and tries to establish acceptable daily intake doses (Vandermeersch et al., 2015). In the US, USEPA published a Contaminant Candidates List (CCL) where it predicts the chemical products that can reach and remain in public waters and proposes and selects products that may require constant monitoring in the future. In 2011, USEPA CCL included pesticides, disinfection by-products, chemical used in commerce, and PhACs, among others (https://www.epa.gov/ccl/contaminant-candidate-list-3-ccl-3). In countries of the European Community, the Parliament regulates the European watch list (European Parliament, 2013) in which diclofenac, 17-β-estradiol and 17-α-ethynylestradiol are included since 2013, and in 2015 so were three antibiotics (azithromycin, clarithromycin, and erythromycin), another natural hormone, estrone (E1), eight pesticides, one UV filter, and one antioxidant compound (Decision 2015/495/EU of 20 March 2015)^1^. The US Food and Drug Administration (FDA) monitors the environmental concentration and requires environmental assessment when the concentration exceeds 1 μg/L to authorize marketing (Bolong et al., 2009; Santos et al., 2010). Also, the ECsafeSEAFOOD database platform (www.ecsafeseafood.eu) is a project that harmonizes and gathers information on the levels and side effects of ECs, including PhACs in seafood by the analysis of more than 400 papers, which allows open access online to that information and includes management systems based on PHP and MySQL (Vandermeersch et al., 2015).

### Structural chemical diversity among pharmaceutical compounds

PhACs cover a wide range of chemical structures. They are usually classified according to their therapeutic function (lipid regulators, anti-inflammatory/analgesic drugs, psychiatric drugs, antibiotics, β-blockers, estrogens, and iodinated contrast media (Cruz-Morató et al., 2012). Largely, they are composed of heterocyclic aromatics containing as heteroatoms nitrogen, oxygen, or sulfur of 3- or 4-fused rings, and macrocycles (Vitaku et al., 2014). The presence of aromatic structures confers to these substances properties related to aromaticity, electrophilic substitution reaction, and resonance stabilization (Gupta et al., 2013) which results in low solubilization, based on their octanol/water partition coefficients. This feature decreases their biodegradability since they are less bioavailable to microorganisms. In addition, in the case of antibiotics, biotransformation with bacteria or fungi could represent a challenge, since the antimicrobial properties hinder their use as carbon source. These aromatic compounds contain molecules bearing electron-donating functional groups such as hydroxyl groups (paracetamol); carbonyl groups (quinolones) and amine groups (carbamazepine, trimethoprim); electronegative chlorine (triclosan, diclofenac), or fluorine groups (ciprofloxacin) in their molecular structures; whilst several molecules are being constituted by the combination of multiple substituents (e.g., diclofenac, diazepam, sulfamethoxazole). Besides the presence of aromatic compounds, we also found linear ones such as carboxylic acids (valproic acid). This vast variety of chemical structures makes it difficult to rule out the behavior and general pathways for microbial transformation.

## The role of fungi in biotransformation and biodegradation of PhACs

Fungi represent one of the most diverse groups of microorganisms, playing key roles in nature as decomposers, mutualists or pathogens (Schmit and Mueller, 2007). The global fungal species richness is controversial since most species of these groups are not yet described (Schmit and Mueller, 2007; Bass and Richards, 2011; Blackwell, 2011), although it is consensual that the majority of this richness involves terrestrial ascomycetes and basidiomycetes (Kirk et al., 2008). Both phyla contain pollutant degraders (Harms et al., 2011). *Mucoromycotina* (formerly *Zygomycota*), a subdivision of fungi of *incertae sedis* (unknown or undefined relationships), represents a noteworthy group less represented than those mentioned above, but which includes some well-studied species that metabolize xenobiotics (Cha et al., 2001; Asha and Vidyavathi, 2009).

**Figure 1:**
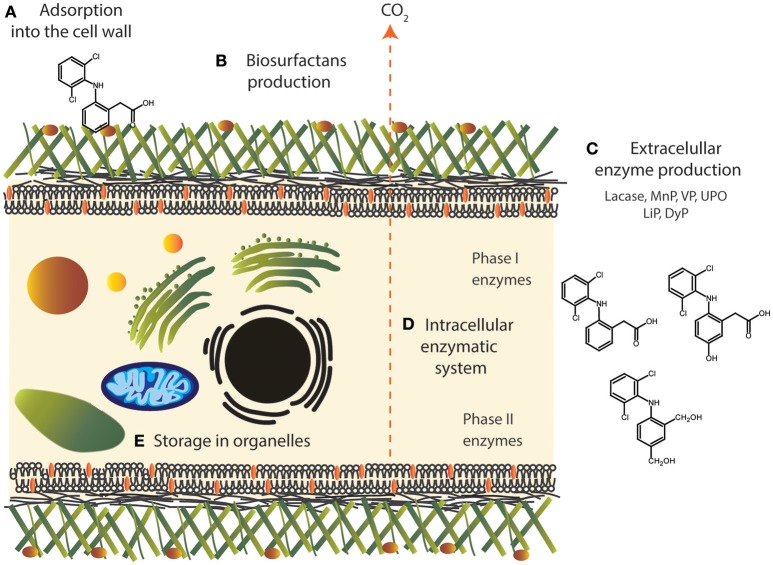
Different mechanisms of fungi to counteract with emerging contaminants, case of diclofenac: **(A)** bioadsorption, **(B)** hydrophobin production, **(C)** metal interaction, **(D)** extracellular enzymatic system, **(E)** intracellular enzymatic system.

Fungi have a variety of strategies to counteract with a myriad of toxic compounds such as recalcitrant polycyclic aromatic hydrocarbons (PAHs) and pesticides (Cerniglia, 1997). These strategies include not enzymatic process such bioadsorption, biomineralization (bio-precipitation) as well as biotransformation and biodegradation mediated by enzymatic systems (Harms et al., 2011) (Figure 1). Bioadsorption is mediated by the specific composition of the cell wall such as chitosan or chitin (Gadd and Pan, 2016). In some fungi, such as *Phoma* sp. UHH 5-1-03, biosorption into fungal mycelia has an important role for bisphenol A, 17α-ethinylestradiol and triclosan removal, until this bioadsorption reaches equilibrium (Hofmann and Schlosser, 2016). In addition, fungi are able to produce biosurfactants, functionally diverse amphiphilic surface active compounds with hydrophilic and hydrophobic portions that interact between phases of different polarities, resulting in interfacial tension reductions and increasing the interaction between molecules (Cicatiello et al., 2016; Günther, 2017). It has been reported that the chemical structure of fungi biosurfactants involve, among others, sophorolipids, protein-lipid/polysaccharide complexes, glycolipids, and glycolipoproteins. These molecules represent an important tool for bioremediation purposes (Bhardwaj et al., 2013). In the case of PhACs, most of them contain aromatic structures, thus, it could be possible that biosurfactants improve the PhACs mobility and increase their bioavailability, as it has been previously observed for PAHs (Souza et al., 2014).

### Biotransformation mediated by enzymes

**Figure 2:**
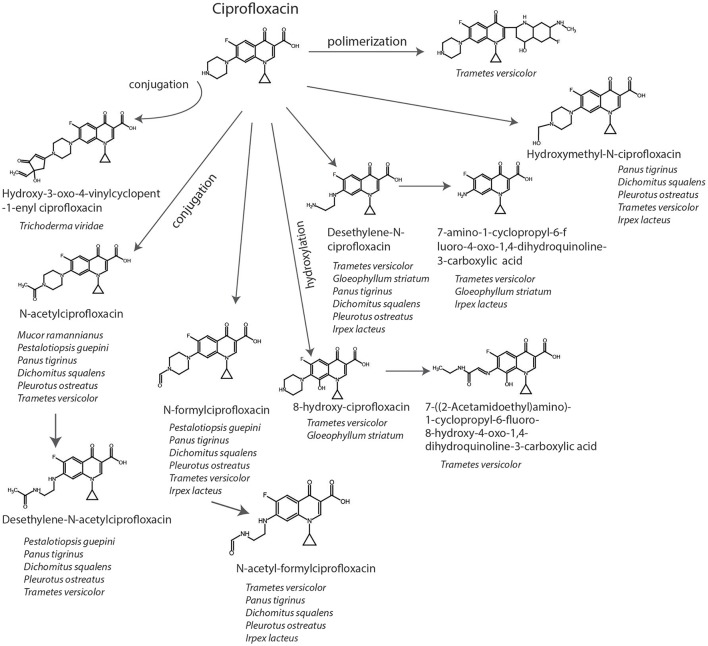
Pathways in the degradation of ciprofloxacin by different phyla of fungi according to Parshikov et al. (2001), Parshikov et al. (2002a), Prieto et al. (2011), and Čvančarová et al. (2015).

Biotransformation of PhACs include hydroxylation, oxidation, sulfoxidation, and dealkylation reactions. Figure 2 represents some of these reactions for degradation of ciprofloxacin by fungi, as an illustration of their potential as bioremediation agents. In some cases, the resulting by-products (so-called dead-end by-products) are further metabolized by other microorganisms, though in some cases they are mineralized to CO_2_ (Badia-Fabregat et al., 2014). Hydroxylation can be regarded as a biotransformation strategy for bioremediation processes, since it can increase the solubility of pollutants and thereby reduce the bioaccumulation potential (Esser, 1986). Some positions of hydroxylation reduce their ecotoxicology, as in diclofenac or flumequine, in which 4-hydroxylation and 7-hydroxylation, respectively, produce metabolites that are less toxic than the parent compounds and suppress antibacterial activity (Williams et al., 2007; van Leeuwen et al., 2011). However, some metabolites produced by fungi such as 1,2-hydroxy ibuprofen can be more toxic than the parent compound (Marco-Urrea et al., 2009). Thus, it is important to consider, before proposing and evaluating a specific system, the possibility that the method could produce a more severe environmental problem than the one it is intended to solve. Hence, ecotoxicity tests at different trophic levels, as well as measures of estrogenic or androgenic activities, including the endocrine interference by means of human cell lines or yeasts (Schilirò et al., 2012; Spina et al., 2015), should be requirements for the implementation of water treatment strategies performed by fungi, since internal complex molecular interaction of different micro-pollutants could have mediator effects. To counteract all of these reactions, fungi possess a machinery of unspecific enzymes and reaction mechanisms that make them highly suited for these types of processes (Harms et al., 2011). The majority of these enzymes are involved in lignin degradation (LME, lignin modifying enzymes) (Kirk and Farrell, 1987) and are including extracellular enzymes, such as class II peroxidases (manganese peroxidase, lignin peroxidase, versatile peroxidase), laccases, and dye decolorizing peroxidases (DyP-type peroxidases). However, the function of some of them, such as unspecific peroxygenases (UPOs) which could have a main role in transformation of PhACs, remain unknown in nature (Hofrichter et al., 2015). These enzymes are able to unspecifically oxidize a wide range of substrates, transferring electrons from organic substrates to molecular oxygen (laccases); by oxidation-reduction reactions using H_2_O_2_ as electron accepting co-substrate (class II peroxidases and DyPs); or by epoxidation, aromatic peroxygenation and sulfoxidation, among others (UPOs) (Karich et al., 2017).

### Intracellular enzymatic system

Although extracellular enzymes and reaction mechanisms play a predominant role in the degradation of PhACs, and the majority of studies have been focused on these enzymes, the internal mechanism of detoxification (Phase I and Phase II) mediated by the cytochrome P450 family (CYP) epoxidases and transferases in coordination or not with the extracellular system has been undervalued. As several authors have stated, an intracellular system is also necessary for transforming different xenobiotics (Guengerich, 2008; Moktali et al., 2012); particularly in fungi characterized by a low secretion of LME -*Ascomycota*- (Aranda, 2016). The hidden involvement of CYP in the transformation of PAHs, hormones, and aromatic compounds has been revealed in different studies (Cerniglia, 1997; Syed et al., 2011; Křesinová et al., 2012; Stella et al., 2013). The cytochrome P450 system is a large family of enzymes, mainly monooxygenases related to several kinds of reactions such as hydroxylation, heteroatom oxygenation, dealkylation, epoxidation of C = C bonds, reduction, and dehalogenation (Díaz-Cruz et al., 2015). They are found in all kingdoms and domains of life and are classified according to amino acid sequence similarity and phylogenetic relationship. *Basidiomycota* species show a considerable number of CYP genes. Some species have around 150 cytochrome P450 genes represented by 12 different groups of these enzymes (Cajthaml, 2015). The comparison of fungal CYPomes with the currently sequenced fungal genomes indicates that fungi have abundant CYPs belonging to diverse families (Chen et al., 2014). However, only 12 P450 clans have been identified in the metabolism of xenobiotics, such as CYP52, CYP53, CYP505, CYP55, among others (Moktali et al., 2012).

**Figure 3:**
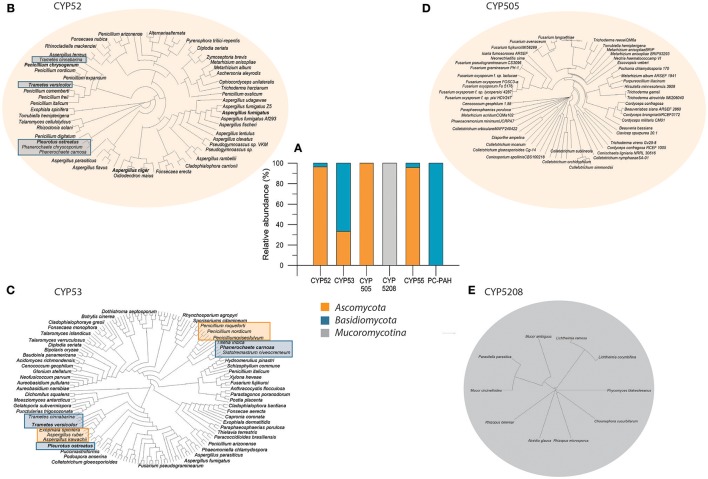
Percentage of relative abundance of some reported Cytochrome P450 enzymes related with fungal biodegradation **(A)** and phylogenetic distribution of CYP52 **(B)**, CYP505 **(C)**, CYP53 **(D)**, and CYP5208 **(E)**, which are involved in xenobiotic transformations, among *Basidiomycota, Ascomycota* divisions and *Mucoromycotina* subdivision. Some fungal species related to the biodegradation of emerging contaminants are highlighted. The phylogenetic distribution is different in each case, depending on the alignments of proteins, according to the abundance of reported CYP in fungi (central graph, **A**). Protein sequences were taken from the database UniProt (Pundir et al., 2015). The BLAST and multiple alignment tools (http://blast.ncbi.nlm.nih.gov/Blast.cgi) were used to detect protein sequence similarities with sequences of fungal species deposited at GenBank. Evolutionary analyses were conducted in Clustal W and Clustal X programs (Larkin et al., 2007) provided by EMBL-EBI bioinformatics (Li et al., 2015a) with neighbor-joining as clustering method. The figure was edited and constructed using MEGA 7 (Hall, 2013), AliView (Larsson, 2014), and Dendroscope 3 (Huson and Scornavacca, 2012) tools.

The distribution of some of these clans associated with the degradation of xenobiotic compounds is illustrated in Figure 3, where it is shown that some clans are associated mainly with *Ascomycota* (CYP52, CYP505, CYP55) (Figure 3A), and others with greater prevalence in *Basidiomycota* (CYP53 and PC-PAH), while the CYP5208 clan is found exclusively in the subdivision *Mucoromycotina*. In particular, analyses of the phylogenetic distribution of these clans, for example CYP52 (Figure 3B), show that the evolutionary distances between basidiomycetes and ascomycetes are similar, indicating a homology in the sequences of the proteins of this clan, reflected in the universal well-characterized functions of the CYPs in both divisions. An example of this strong similarity between the CYPs of clan 52 is *T. versicolor*, which has a closer evolutionary relationship with *Penicillium* species than with *T. cinnabarina*, although it is a clan described mostly for *Ascomycota*. In the same line, the CYP53 clan (Figure 3C) is present in *Ascomycota* and *Basidiomycota* with a similar phylogenetic distribution, although with more random divergent points. Clans CYP505 and CYP5208 (Figures 3D,E) and the PAH-inducible cytochrome P450 monooxygenase family are reported only in *Ascomycota, Mucoromycotina* and *Basidiomycota*, respectively. The study of the CYP distribution as well as genetic homology could shed light on the capability of different fungi to deal with PhACs and their potential for bioremediation purposes and therefore these findings could help to better design strategies for PhACs transformation.

### Other mechanisms

Reaction mechanisms include Fenton reactions, with redox cycling of quinones being the most studied ones. This mechanism may or may not be mediated by LME, and thus, this mechanism is suspected to be widely distributed throughout the fungal kingdom (Guillén et al., 2000; Krueger et al., 2016). The non-specificity and the high oxidation potential of hydroxyl radicals generated in this reaction make them a powerful tool for the efficient degradation of a broad range of pollutants (Gómez-Toribio et al., 2009). In the fungus *T. versicolor* this system has been studied for a battery of PhACs (clofibric acid, carbamazepine, atenolol, and propanolol), and hydroxyl metabolites appear with the induction of oxidizing agents, as in the case of the metabolites found in photocatalytic wastewater treatments (Marco-Urrea et al., 2010b).

The combination of all of these mechanisms provides an integral view of the versatility for applying fungi. The mechanisms heterogeneity and the variety of species makes it difficult to involve just one single method for the elimination of ECs. Thus, the new approaches should consider more than one way to solve the problem and implement an efficient system by applying the appropriate strains of fungi, according to the intrinsic characteristics of the wastewater, and by integrating these approaches in the current wastewater treatment systems.

## *Basidiomycota* fungi

The *Basidiomycota* division of fungi is a group of macro- and microorganisms characterized by the formation of basidia, a bottle-shaped cell structure containing haploid and sexual basidiospores. During their life cycle, most *Basidiomycota* fungi pass between a dikaryotic state and diploid growth to asexual reproduction by conidia with the subsequent formation of basidiospores (Alexopoulos et al., 1996). Due to their environmental relations, *Basidiomycota* fungi have developed and optimized different systems related to the cycling of carbon and nitrogen sources, as well as to ecosystem balance. This provides them with a series of extracellular and intracellular mechanisms capable of interacting with heterogeneous substrates, mainly lignin derivatives (Schmidt-Dannert, 2016). One of the most attractive reasons for the use of *Basidiomycota* fungi in the degradation of PhACs is the great variety of substrates that they can metabolize (Maciel et al., 2010).

The main mechanism in the degradation of PhACs and aromatic compounds by *Basidiomycota* is the use of the above-mentioned groups of extra- and intracellular oxidoreductases (laccases, peroxidases, CYPs, etc.) to modify and break down the bonds of different compounds, mainly by extracellular pathways (Schmidt-Dannert, 2016). In this context, several kinds of LME and fungal mediators have been studied in relation to the biodegradation of PhACs in the white rot fungi *Phanerochaete chrysosporium, Phlebia ochraceofulva, Pycnoporus sanguineus, Pleurotus ostreatus*, and *T. versicolor* (Cajthaml et al., 2009; Díaz-Cruz et al., 2015). However, in all cases the participation of the CYP has a pivotal role on PhACs transformation, usually determined by indirect measurements with CYP inhibitors such as 1-aminobenzotriazole and piperonyl butoxide.

### Whole cell fungal treatments

The current approach of the treatment of PhACs in wastewater using this kind of fungi is the development of systems that could be implemented in real life by the design and improvement of bioreactors and the use of real wastewater from municipal, industrial or hospital effluents. From this perspective, several studies have focused on the use of whole cell *Basidiomycota* fungi, especially *T. versicolor*, to optimize degradation conditions as well as to implement new techniques for the monitoring of PhACs (Badia-Fabregat et al., 2014, 2015; Llorca et al., 2015; Picó and Barceló, 2015). Evaluations have been made of the removal rate of PhACs (Marco-Urrea et al., 2010a; Cruz-Morató et al., 2013; Ba et al., 2014) or endocrine disruptors (Catapane et al., 2013; Cruz-Morató et al., 2014; Ferrando-Climent et al., 2015) using free *T. versicolor* cells in fluidized-bed, batch, and membrane bioreactors under sterile and non-sterile conditions, in which laccases and CYPs are involved (Cruz-Morató et al., 2013, 2014; Yang et al., 2013). The results are satisfactory, with 70–100% effectiveness. However, the heterogeneous composition of real wastewater makes it difficult to implement systems capable of degrading all toxic substances or achieving 100% removal for all of them (Cruz-Morató et al., 2013). In addition, it is also necessary to evaluate not only the capability to remove PhACs, but also the ability to produce a consistent COD and toxicity reduction of the resulting effluents, as well as the capability of the fungus to coexist with autochthonous microorganisms present in the current biological systems. In some of these studies, a toxicity reduction has also been achieved; however, no comparisons on the efficiency on the reduction of COD in relation to conventional treatments were given. In addition, under non-sterilized conditions the introduced fungus was displaced by the native fungal and bacterial population (Badia-Fabregat et al., 2017).

Although *T. versicolor* is the basidiomycete most commonly used in the biodegradation of PhACs, there is evidence of the use of other models in combination with different removal techniques. For example, heterogeneous catalytic processes have been described with *P. ostreatus* and the use of γ-Fe_2_O_3_ nanoparticles in bisphenol A degradation, reaching a removal rate 32% greater with the combination of biotransformation and Fenton-like reactions under oxidant conditions (Li and Zhang, 2016). The possibility of releasing white rot fungi and oxidative nanoparticles in the environment constitutes one of the advantages of having a practical system to be applied. Likewise, in the same model, the biotransformation of carbamazepine in liquid culture has been compared with solid-state fermentation on lignocellulosic substrate by evaluating the metabolic products. The results revealed that the metabolic pathways differed in both cases, indicating the generation of two metabolites in submerged fermentation and 24 metabolites in solid-state fermentation, some of which being more toxic than the parent compound (Golan-Rozen et al., 2015). Thus, it is important to consider that the environmental conditions have a vital influence on the degradation of the PhACs, since LME are inducible by different factors. For example, comparisons were made on the efficiency of the elimination of β-estradiol by laccases from *Pycnoporus* sp., *T. versicolor* and *Hymenochaete spreta* cultured in solid state with the addition of citric acid and lignocellulosic biomasses to optimize enzyme production. The addition of these substances improved to different levels the secretion of laccases and enabled estradiol elimination up to 80% (Liu et al., 2016). However, these inductors-linked degradation could have an economic negative impact in real application as well as consequences in the operational conditions (for instance release of dark pigments).

The use of whole cell biotransformation has the advantage that all the fungal mechanisms (enzymatic or not) could take part in the process. For example, it was observed that the use of whole cell of *Phoma* sp., improves the elimination of a greater variety of compounds (including diclofenac and carbamazepine) compared with the enzymatic extract (Hofmann and Schlosser, 2016). However, the filamentous growth could have operational problems associated (clogging, fouling and problems for biomass separation). Thus, the current approach of the use of fungi is to establish the best conditions for the degradation of PhACs in real wastewater and under environmental conditions, as well as to find a profitable balance of the costs of the operation systems implemented in each case.

### Enzymatic transformation using LMEs

The use of purified and crude extracts of enzymes such as laccases, manganese peroxidases, and versatile peroxidases have also been studied for PhACs removal, as has been reviewed by Cruz-Morató et al. (2012), with high rates of effectiveness of transformation.

The possibility to avoid the purification step could reduce costs and improve the adaptability; however, in some cases, high amounts of crude extract should be employed to achieve desirably high rates of conversion. The crude extract containing manganese peroxidase from *Phanerochaete chrysosporium* was evaluated in the elimination of tetracycline and oxytetracycline with removal rates of 72.5 and 84.3%, respectively, in an average degradation time of 4 h (Wen et al., 2010).

However, the use of purified enzymes allows a better control of the process and high rates of conversion of the compound in question. Several examples showed high rates of effectiveness of transformation after short reaction times with single enzymes of 10–60 min. Also, the use of purified and combined free cross-linked enzyme aggregates composed of laccase from *Trametes versicolor*; versatile peroxidase from *Bjerkandera adusta*, and glucose oxidase from *Aspergillus niger* were characterized in the elimination of PhACs. The cross-linked mix proved stability under environmental and denaturing conditions in the elimination of acetaminophen, naproxen, mefenamic acid, indometacin and diclofenac, among others, with elimination rates greater than 80% (Touahar et al., 2014). The implementation of immobilized laccases and peroxidases from different basidiomycetes have been tested vs. several PhACs with considerable removal rates between 60 and 100% (Macellaro et al., 2014b; Li et al., 2015b).

Enzymatic activities can be improved by the use of fungal redox mediators. Some examples of using endocrine-disrupting compounds (bisphenol A, nonylphenol, methylparaben, butylparaben, and dimethylphthalate) can be found, using different laccase enzymes from *P. ostreatus* (ATCC number MYA-2306). The influence of a synthetic (2,2′-azino-bis (3-ethylbenzothiazoline-6-sulphonic acid, ABTS) and natural (acetosyringone, AS) fungal mediators were evaluated regarding the degradation efficiency of these enzymes, by boosting the degradation rate by around 50%. However, in some kinds of laccases the influence of fungal mediators did not significantly improve the removal rate, indicating that a simple rule cannot be established since the mechanisms of enzymatic affinity can be very complex and vary from one species to another (Macellaro et al., 2014b). In addition, the toxicity of synthetic fungal mediators is a disadvantage for the proposal of real bioremediation systems using these substances (Margot et al., 2015; Chen et al., 2016). UPOs have also been investigated in the transformation of a battery of ECs with the goal of synthesizing hydroxylated and O- or N-dealkylated human drug metabolites, reactions which can be applied as a tool for bioremediation purposes (Kinne et al., 2009; Poraj-Kobielska et al., 2011).

The main challenges to overcome in these systems are to prolong enzyme life, maximize the enzyme reuse capacity, and improve enzyme viability to immobilization and implementation conditions. Thus, new approaches using genetic engineering tools can improve these parameters. The use of magnetic derivatives of natural matrixes such as chitosan or biotin combined with LME enzymes has also shown high levels of re-use rates (90% of the activity retained after five reaction cycles and 60% after 10 cycles), and improved enzyme recovery systems vs. PhACs such as bisphenol A, 17α–ethinylestradiol, and diclofenac mixtures (Ardao et al., 2015).

Other aspects that improve the removal rate of PAhCs are the mixture between LME enzymes and nanoparticles, in order to generate a stable and flexible matrix for the elimination of pollutants. Using magnetic laccase-biotitania particles (lac-bioTiO_2_) with the laccase from the ascomycete *Thielavia* sp. and an immobilization by adsorption phenomes and biotin precipitation, an evaluation was made for the biodegradation of endocrine disruptors (bisphenol A, 17α ethinylestradiol) and diclofenac, which retained 90% of activity after five reaction cycles and 60% after 10 cycles, thus improving enzyme reuse (Ardao et al., 2015).

## *Ascomycota* fungi

*Ascomycota* constitutes the most variable phylum of the fungal kingdom, accounting for >65% of the currently described fungi (Harms et al., 2011). This diverse group includes microorganisms that can exist in both reproductive states (anamorph and teleomorph), making their classification extremely difficult (Hibbett and Taylor, 2013). This phylum contains all the life styles (parasitic, symbiotic or saprotrophic) and morphologies: unicellular (yeast), multicellular (filamentous), and dimorphic fungi (can exist as mold/hyphal/filamentous form or as yeast). In addition, they are able to colonize very diverse niches. The biodiversity of fungal communities in activated sludge or wastewater-treatment plants has been largely disregarded (Weber et al., 2009; Tigini et al., 2014). Analyses of the fungal diversity in anthropogenically polluted samples, such as activated sludge or wastewater-treatment plants indicate *Ascomycota* as the dominant phylum (Weber et al., 2009; Evans and Seviour, 2012; Maza-Márquez et al., 2016). Their high adaptation is supported by the fact that they have a number of advantages in these environments, such as the capability to chelate metal ions (leading to detoxification), which are very frequent in wastewater (Gadd et al., 2014; Tigini et al., 2014). Further the ability to grown fast at even slightly basic pH values, and the power to resist adverse conditions (Harms et al., 2011). These capabilities were evidenced when experiments in bioreactors were performed with non-sterile wastewater using *T. versicolor*. After 7–15 days in an unsterile bioreactor, this fungus lost its predominance while species from *Ascomycota* such as *Fusarium* and *Trichoderma* from them dominated the bioreactor, now strongly contributing to the degradation of PhACs (Badia-Fabregat et al., 2017).

Despite this wide distribution, the use of *Ascomycota* for PhACs degradation has been poorly studied. The degradative capability of this group of fungi has been studied in aromatic substances such as PAHs, chlorinated hydrocarbons, and diverse xenobiotic compounds (Marco-Urrea et al., 2015; Aranda, 2016; Bovio et al., 2017). They are characterized by the involvement of the intracellular enzymatic system mediated by CYP, and some species are also able to secrete LMEs such as manganese peroxidase or laccase. In this context, the entire complexity of the metabolic pathway of conversion is not well established. Some hardly explored enzymes such as UPO could also play a main part in extracellular hydroxylation. In this sense, it is estimated that UPOs are widely distributed in *Ascomycota*, which, as previously mentioned, are known to perform a broad range of enzymatic reactions on aromatic compounds and drug metabolites (Poraj-Kobielska et al., 2011; Hofrichter et al., 2015).

For this group of fungi, as opposed to *Basidiomycota*, no model organism is available to study PhACs degradation, and its application in real bioprocesses remains remote.

Degradation of non-steroidal-anti-inflammatory drugs (NSAIDs), such as diclofenac, have been studied using *Epicoccum nigrum* (IMI3542), which is able to catalyze the complete conversion of diclofenac to 4-hydroxydiclofenac (90%) (Webster et al., 1998). However, other species such as *Pestalotiopsis* sp. (IMI353656) cause hydroxylation in positions 3, 5, and 4. Fungi such as *Aspergillus nidulans* and *Bipolaris tetramera* can produce several hydroxylated compounds from diclofenac, although the authors did not discriminate the position of the hydroxyl group (Gonda et al., 2016).

The degradation of antibiotics such as fluoroquinolones and quinolones have been studied by different representatives of *Ascomycota*. A preliminary experiment was performed by Zhang et al. (2012), who isolated from a soil artificially contaminated with the antibiotic ciprofloxacin the strains *Penicillium notatum, Aspergillus fumigatus, Penicillium frequentans*, and *Penicillium expansum*. The authors detected a decreased amount of ciprofloxacin; however, the metabolites that formed were not analyzed in these experiments. *Trichoderma viride* produced conjugated compounds when incubated with ciprofloxacin and norfloxacin, producing hydroxy-3-oxo-4-vinylcyclopent-1-enyl ciprofloxacin and 4-hydroxy-3-oxo-4-vinylcyclopent-1-enyl norfloxacin (Parshikov et al., 2002a), which has less antibacterial activity than does the parent compound (Zeiler et al., 1987). The fungus *Beauveria bassiana* can transform cinoxacin by reducing a carboxyl group to hydroxymethyl and to cleave a dioxolo ring (Parshikov et al., 2002b). These results indicate that we cannot find a general pattern in the pathways of quinolone transformation by ascomycetes, despite the fact that the general mechanism of the detoxification of xenobiotic by these fungi is mediated by CYPs.

Although ascomycetes could be able to degrade a large number of compounds, the complexity and variability of PhACs require the use of alternative methods to increase the removal rates.

The study of native fungi should be prevalent instead of the use of allochthonous microorganisms for bioremediation purposes. Firstly, because they are well adapted, and secondly, because the different countries have specific protective laws and regulations, in order to minimize potential environmental perturbations (El Fantroussi and Agathos, 2005; Federici et al., 2007). This fact represents a motivation for the study of ascomycetes. In recent years, the use of fungal consortia using autochthonous species belonging to *Ascomycota* has been built and marketed at an industrial level to improve the biodegradation of several contaminants (Mishra and Malik, 2014). Some fungi commercially used in wastewater treatment of dairy wastewater that resulted in a complementary and synergistic effect for the reduction of contaminants include *A. niger* and *Galactomyces geotrichum*, combined with the mucormycete *Mucor hiematis* (Djelal and Amrane, 2013). In addition, other species reported to form part of consortia involved in the contaminant removal are *Cladosporium perangustum, Penicillium commune, Paecilomyces lilacinus*, and *Fusarium equiseti* (Sharma and Malaviya, 2016), isolated from soils contaminated with tannery wastewater. These fungi have shown a high efficiency in industrial wastewater biotransformation, being able to remove a myriad of compounds, some of which were included in ECs. This fact opens the possibility to use these consortiums in pharmaceutical wastewater treatments.

## *Mucoromycotina* fungi

*Mucoromycotina incertae sedis* (formerly *Zygomycota*) represents a heterogeneous group which monophyli is currently under discussion. They are characterized by the formation of zygospores in the sexual phase and aplanospores in the asexual phase (Benny et al., 2014).

**Figure 4:**
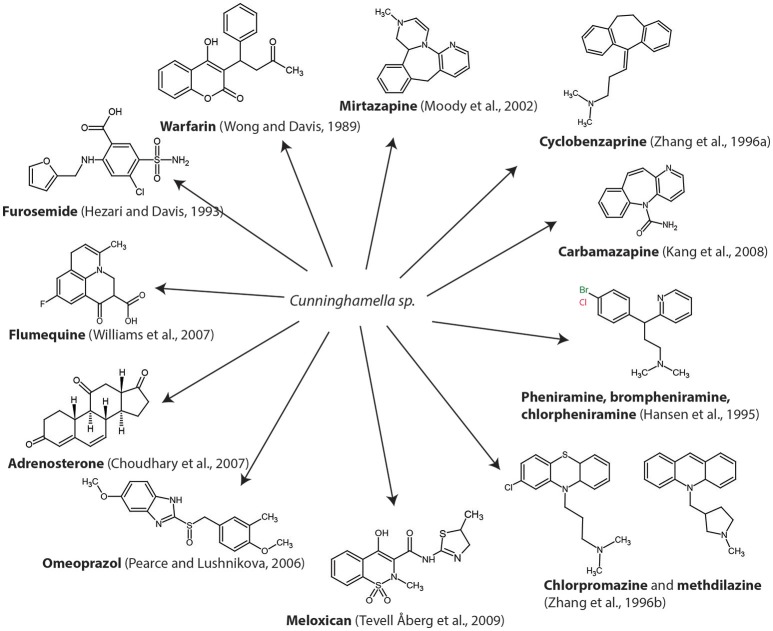
Spectra of pharmaceutically active compounds were studied in *Cunninghamella elegans* by different authors.

Among all the life styles present in this group (saprophytes, mutualists, and pathogens), members of *Mucorales* which are the core group of the traditional *Zygomycota*, are saprobes or facultative parasites in nature. Some members *Mucorales* such as *Cunninghamella elegans* have been extensively used as model fungi in different studies on the metabolism of xenobiotics, due to their ability to produce regio- and stereo-selective transformations, as in mammal enzymatic systems. Thus, they represent a tool for emulating mammal drug metabolism and for manufacturing metabolites of industrial interest (Asha and Vidyavathi, 2009). The high capability of this fungus to transform a broad spectrum of compounds has been studied in lab scale and are represented on Figure 4 such as the diuretic furosemide (Hezari and Davis, 1993), the anti-inflammatory meloxican (Tevell Åberg et al., 2009), antibiotics such as fluoroquinolones (flumequine) (Williams et al., 2007), the pro-hormone adrenosterone (Choudhary et al., 2007), the anti-gastroesophageal reflux omeoprazol (Pearce and Lushnikova, 2006), the anticoagulant warfarin (Wong and Davis, 1989), antihistamines such as brompheniramine, chlorpheniramine, and pheniramine (Hansen et al., 1995), antipsychotics such as chlorpromazine and methdilazine (Zhang et al., 1996b), the muscle relaxer cyclobenzaprine (Zhang et al., 1996a), the anti-depressant mirtazapine (Moody et al., 2002) or the anticonvulsant carbamazepine (Kang et al., 2008). The biotransformation of PhACs by *C. elegans*, in general, undergo a phase I reaction (oxidation, reduction, and hydrolysis), generating hydroxylated metabolites, (2-hydroxycarbamazepine, hydroxyflumequine, hidroxywarfarin, etc.) and sulfoxidated products (chlorpromazine sulfoxide). These reactions are highly regio- and stereoselective. In some cases, these compounds undergo phase II reactions to form conjugated metabolites (fluoresomidearylglucoside).

Other representatives of *Mucoromycotina* such as *Umbelopsis ramanniana* and *Mucor rammanianus* have been investigated in microbial transformations of carbamazepine (Kang et al., 2008) and fluoroquinolones, such as sarafloxacin and enrofloxacin, respectively (Parshikov et al., 2000, 2001). Also, in *C. elegans*, these compounds initially undergo diverse monooxygenation reactions leading to the formation of epoxides and ring hydroxylated metabolites (3-hydroxycarbamazepin). Fluoroquinolones, in these representatives, undergo N-oxidation, N-dealkylation, and N-acetylation reactions which have less antibacterial activity than the parent compounds (Parshikov et al., 2001).

Despite the potential of this group of fungi for removal of several ECs, the scaling-up to real-life conditions has not yet been developed. Thus, the behavior of this species in real wastewater in terms of COD reduction, as well as its competition capability against other microbial species remains still unknown.

## Perspectives of fungal genetic engineering toward transformation of PhACs

Genetic engineering could represent an essential step to improve the bioremediation by fungal systems, to modify the enzyme activities and affinities of target compounds and to develop new techniques for fungal adaptation. This requires the development of robust genetic modification methods to obtain fungi able to interact with a greater number of contaminants, reduce toxic degradation by-products, and generate efficient systems that can be implemented (Joutey et al., 2013). Also, the possibility of having all the advantages of a consortium in one microorganism is an attractive option, providing simplicity and reducing operating costs (Joutey et al., 2013).

From the point of view of enzyme overexpression, laccases, peroxidases, cellulases, and ligninases from white-rot fungi expressed in host *Ascomycota* such *Aspergillus* and *Penicillium*, has generated a large number of research papers as well as patents around the world (Beijersbergen et al., 2001; Shakeri et al., 2007; Ward, 2012; Cortés-Espinosa and Absalón, 2013; Macellaro et al., 2014a). However, its implementation is currently being tested to have an efficient system for the removal of different compounds (Mekmouche et al., 2014; Antošová and Sychrová, 2016).

Focused on the application of genetic engineering for ECs removal, some examples of the application of recombinant enzymes can be found within the expression of LMEs, such as a chimerical laccase of *Pleurotus eryngii* in *Saccharomyces cerevisiae*, which reportedly improved the activation, affinity, and activity of the enzyme, by N- or/and C-terminus modification. The use of recombinant enzymes has been tested in the elimination of hydroxylated polychlorinated biphenyls, with removal rates between 10 and 60%, depending on the congeners used (Fujihiro et al., 2008; Piscitelli et al., 2010). Also, the immobilization method by the expression of laccase into the cell wall of the host represents an innovative tool, which allows a common and rapid growth of yeast (Bleve et al., 2014). In this context, yeast surface display-recombinant laccase for the biotransformation of bisphenol A and sulfamethoxazole, with 80 and 40% as a removal rate, respectively; with an increase in removal efficiency in the presence of enzymatic inductors and 90% of reuse efficiency after 25 cycles (Chen et al., 2016). Successful results can be found with recombinant laccases from the basidiomycete *Pycnoporus cinnabarinus* expressed in *Aspergillus oryzae* and *A. niger* by the inclusion of *lacI* gene, in order to develop a production process without using laccase inducers required by the native enzyme (Sigoillot et al., 2004). The native and the two recombinants enzymes were found to share physicochemical and biochemical properties which made it possible to establish a method to produce this enzyme by heterologous gene expression in *Aspergillus*. This fungus also offers the advantage of having a complex intracellular system for degrading contaminants and good adaptation, resulting in a modified microorganism attractive for bioremediation of PhACs (Corso and Maganha de Almeida, 2008; Vatsyayan et al., 2008; Lubertozzi and Keasling, 2009). The information for the application under real environmental conditions of these microorganisms is insufficient and limited; so, the next step is to develop systems in which these genetically modified microorganisms (GMOs) can be tested for the elimination of PhACs, taking into account the limitations established by the legislation on the deliberate use of GMOs. The laccase of *Trametes sanguineous* has been also overexpressed in *Trichoderma viride*, a strain which showed a better capability for the removal of bisphenol A than the wild-type (Balcázar-López et al., 2016). Manganese peroxidase was also expressed in *Aspergillus* to improve its potential as bioremediator. Thus, *A. niger* was modified by the use of a transforming cassette that included the manganese peroxidase gene (*mnp1*) from *P. chrysosporium*, the constitutive promoter glyceraldehyde phosphate dehydrogenase, *gpdA* from *A. nidulans*, and the glucoamylase terminator of *A. awamori* to improve PAH degradation by the expression of this enzyme. Despite the fact that systems have not been applied for the degradation of PhACs, the new strain can reportedly degrade PAHs in high concentrations compared to other ligninolytic and non-ligninolytic fungal strains (Cortés-Espinosa and Absalón, 2013), opening the possibility of application to other aromatic compounds such as PAhCs.

The mentioned modification of fungi includes the presence of a host able to express the protein of interest, the transforming vector, the technology for DNA integration (by for example using *Agrobacterium tumefaciens*), and methods that allows the screening of transformed strains (Haon et al., 2015) obtaining successful recombinant fungi in *Ascomycota, Basidiomycota, Mastigo*- and *Mucoromycotina* (Beijersbergen et al., 2001; Ward, 2012). However, the disadvantages of transforming filamentous fungi include changes in post-transcriptional treatment of recombinant proteins, resulting in low activity, defects in the morphology, low frequencies of transformation (Wang et al., 2005; Ward, 2012). Probably, the development of techniques of genome editing such as CRISPR-Cas9 (clustered regularly interspaced short palindromic repeats) in fungi could solve these problems in a near future and allow the use of filamentous fungi at real scale for the bioremediation of PhACs. This technique could allow over expression of tailored enzymes for bioremediation, like the overexpression of cellulases in *Myceliophthora* strains (Liu et al., 2017). Thus, in some cases, the solution for bioremediation processes based on fungi is the selection of the appropriate microorganism suited for the specific conditions and the stimulation of the specific enzymatic machinery coupled to the degradation of another pollutant that could act as stimulator (Spina et al., 2016).

Finally, although it is evident that genetic tools are important steps in developing new microorganisms that allow the use of adequate bioremediation systems and that the effort to sequence the genome of fungi would allow the development of new genetic techniques for improving the adaptive and metabolic processes, the risks involved in using genetically modified microorganisms and their impact on the environment and human health should not be overlooked. Therefore, the use, manipulation, and implementation of systems that include these types of microorganisms should be carefully analyzed. Also, a new model for legislation about the use of genetically modified organisms should be a priority in all countries for implementation of more efficient systems with adequate control for the coexistence of all microorganisms involved in bioremediation and reduce the impact that the use of GMOs can generate.

## Conclusions

Fungi belonging to *Basidiomycota, Ascomycota* and *Mucoromycotina* can remove and transform different pharmaceutical compounds at different rates, including antibiotics, psychiatric-, anticonvulsant-, and anti-inflammatory drugs or estrogens, among many others, producing different hydroxylated, conjugated and oxidized metabolites. This finding reveals pathways similar to those of other aromatic xenobiotic compounds, indicating that PhACs become vulnerable to enzymatic attack and other mechanisms of fungal transformation. Despite the well-known involvement of CYP enzymes in this transformation, there is still a higher prevalence to use cell-whole white-rot fungi against non-LME-producers, as well as to focus all the genetic engineering on improving the secretion of LME enzymes in these systems, partly because of the great advantages of extracellular enzymes and partly because of the lack of studies on ascomycetes and mucormycetes. Additionally, many gaps in enzymatic conversion and full-conversion pathways remain unresolved, hampering the use of fungi with this purpose. Genetic engineering could help to counteract this mismatch, which represents an essential step forward to improve bioremediation by fungal systems. However, it is first necessary to develop efficient genetic-manipulation strategies validated for all the fungi. However, this represents an obstacle for many fungi of bioremediation interest. In a step forward, a few studies using fungi are coupled to a set of ecotoxicological tests and estrogenic and androgenic experiments, the main concern being to verify the applicability of these processes and to study the fate of the metabolites produced, particularly conjugated metabolites and quinones.

The main goal on bioremediation involves further application in real-life processes, although, from this viewpoint, fungal bioremediation is in its infancy. Ascomycetes and mucormycetes could represent betters competitors than basidiomycetes, not only for their high tolerance to extreme conditions, but also because the distribution of CYP in the annotated fungi reveals a high presence of CYPs involved in the metabolism of xenobiotics in both groups, especially in ascomycetes. Another issue is the implementation and evaluation of pilot plants in order to improve the use of filamentous fungi for bioremediation at the industrial scale. In this sense, the majority of the studies using ascomycetes and mucormycetes have not been scaled-up. Thus, many studies on the physiology and functional genomics in representatives of different groups still need a thorough understanding of the mechanisms for the metabolism of PhACs, particularly in the case of ascomycetes, in order to create a powerful tool applicable in wastewater-treatment plants in order to prevent PhACs from entering ecosystems.

## Author contributions

DO wrote the manuscript (sections Pharmaceutical Compounds in the Environment, The Role of Fungi in Biotransformation and Biodegradation of PhACs, Biotransformation Mediated by Enzymes, Intracellular Enzymatic System and Basidiomycota Fungi, and Perspectives of fungal genetic engineering toward transformation of PhACs, Table 1 and Figure 3). JG helped write and design the manuscript and contributed critical comments on the total of the manuscript. EA designed and wrote the manuscript (Abstract, sections Pharmaceutical Compounds in the Environment, Mucoromycotina fungi and Conclusions, The Role of Fungi in Biotransformation and Biodegradation of PhACs, Intracellular Enzymatic System, Other Mechanisms, Ascomycota Fungi, and Figures 1, 2, 4) and edited and compiled the overall of the text.

### Conflict of interest statement

The authors declare that the research was conducted in the absence of any commercial or financial relationships that could be construed as a potential conflict of interest. The reviewer LYW and handling Editor declared their shared affiliation, and the handling Editor states that the process nevertheless met the standards of a fair and objective review.

## Footnotes

^1^Commission Implementing Decision (EU) 2015/495 of 20 March 2015 establishing a watch list of substances for Union-wide monitoring in the field of water policy pursuant to Directive 2008/105/EC of the European Parliament and of the Council (notified under document C(2015) 1756).
